# Eco-Friendly Facile Conversion of Waste Eggshells into CaO Nanoparticles for Environmental Applications

**DOI:** 10.3390/nano14201620

**Published:** 2024-10-10

**Authors:** Kathalingam Adaikalam, Sajjad Hussain, Periasamy Anbu, Arulmozhi Rajaram, Iyyakkannu Sivanesan, Hyun-Seok Kim

**Affiliations:** 1 Millimeter-Wave Innovation Technology (MINT) Research Center, Dongguk University-Seoul, Seoul 04620, Republic of Korea; kathu@dongguk.edu; 2 Department of Nanotechnology and Advanced Materials Engineering, Sejong University, Seoul 05006, Republic of Korea; shussainawan@gmail.com; 3 Center for Global Health Research, Saveetha Medical College & Hospital, Saveetha Institute of Medical and Technical Sciences (SIMATS), Saveetha University, Thandalam, Chennai 602105, Tamil Nadu, India; anbu25@yahoo.com; 4 Department of Chemistry, SRM Institute of Science and Technology, Kattankulathur 603203, Tamil Nadu, India; arulmozr@srmist.edu.in; 5 Department of Bioresources and Food Science, Institute of Natural Science and Agriculture, Konkuk University, Seoul 05029, Republic of Korea; isivanesan@gmail.com; 6 Division of Electronics and Electrical Engineering, Dongguk University-Seoul, Seoul 04620, Republic of Korea

**Keywords:** calcium oxide, waste eggshell utilization, calcination, photocatalyst, methylene blue dye, dye degradation, antibacterial activity

## Abstract

Amongst the many types of food waste, eggshells contain various minerals and bioactive materials, and they can become hazardous if not properly disposed of. However, they can be made useful for the environment and people by being converted to environmentally friendly catalytic materials or environmental purification agents. Simple calcination can enhance their properties and thereby render them suitable for catalytic and environmental applications. This work aimed to prepare CaO from waste eggshells and examine its effectiveness in photocatalytic pollution remediation, electrocatalytic activity, optical sensing, and antibacterial activities. As opposed to other techniques, this calcination process does not require any chemical reagents due to the high purity of CaCO_3_ in eggshells. Calcium oxide nanoparticles were prepared by subjecting waste eggshells (ES) to high-temperature calcination, and the synthesized CaO nanoparticles were characterized for their structural, morphological, chemical, optical, and other properties. Furthermore, their photocatalytic degradation of methylene blue dye and antibacterial efficiency against *Escherichia coli* and *Staphylococcus aureus* were investigated. It was found that the green-converted CaO can be efficiently used in environmental applications, showing good catalytic properties.

## 1. Introduction

Food waste is increasing rapidly with the increase in the global population, and it is posing a challenge to the environment. The improper management of food waste poses serious problems to the public and environment. Among various types of food waste, chicken eggshells contribute a major portion, since chicken eggs are used invariably among all people and in all food industries and restaurants. Since they are rich in protein, vitamins, and minerals, chicken eggs are commonly used as food worldwide in restaurants, bakeries, and households [1]. Chicken eggshells are discarded as waste by food industries, hotels, and homes. The commonly used disposal method for eggshells is in landfills, and a huge amount of eggshells are discarded in landfills worldwide. Every day, large quantities of eggshells are discarded as waste, which causes serious environmental issues indicating the need for the proper disposal of waste eggshells [2]. Although they can be decomposed on land and used as manure, their biodegradation is time-consuming and has undesirable effects: it promotes the growth of worms and insects and produces odors.

Therefore, this eggshell waste can create environmental degradation, causing human health issues. The conversion of this waste as a useful and marketable product is needed to meet economic and ecologic interests [3]. The main content of eggshell is CaCO_3_, and it can be used for many industrial purposes. CaCO_3_ can also be easily converted into CaO and used in various applications [4]. Moreover, eggshells are a source of calcium and can be used to produce different calcium salts as an alternative to calcium carbonate. Like CaO, many other useful materials like calcium phosphate bioceramics and hydroxyapatite can be produced from eggshells as implanted constituents for a variety of applications [5]. Hence, instead of discarding eggshells as waste, if we convert them into CaO nanoparticles through green routes [6], they can be efficiently used for a variety of purposes, which include agricultural use. Using eggshells for producing CaO can not only help clean the environment but also provide a highly efficient material that can be used for a variety of applications [7,8,9].

Nanoparticles (NPs) of metal oxides can be used for a variety of applications in different fields [10]. Among various metal oxides, calcium oxide (CaO) is an alkali-earth metal oxide [11] that is considered promising for a variety of applications owing to its multifunctional characteristics. CaO NPs can be used for many purposes, such as in CO_2_ adsorption and water purification and as antimicrobial agents [12]. They are excellent adsorbents of gases such as CO_2_ and SO_2_ [13], and they are also used as an additive in the manufacture of paints and in various biomedical applications. In the medical field, they are used for applications such as drug delivery and phototherapy and as chemotherapeutic agents. As they are efficient carbon capturing agents, they can be used as an efficient low-cost pollution remediation agent. They can be easily converted into CaCO_3_ under normal conditions by promoting their adsorption of CO_2_. One of the most important advantages of CaO NPs is that they are easy to produce at low cost.

Nanoparticles are widely used for the treatment of microbial infections owing to their high antimicrobial activity. Studies have demonstrated that NPs can effectively inhibit a diverse range of bacteria, including antibiotic-resistant strains, making them valuable in addressing modern infection challenges [14,15]. CaO NPs have distinctive catalytic and biological properties, which are highly influenced by the morphology of the nanoparticles. NPs incorporated into cloths and food materials are becoming attractive to reduce bacterial growth and related infection problems. Inorganic CaO NPs have stronger antimicrobial activity than organic antimicrobial agents. They are also used as electrochemical catalysts. Catalytic materials are largely required in applications involving electrochemical reactions. A variety of catalysts are used in electrochemical applications, and among them, metal oxide catalysts form an important class of catalysts. Most metal oxide catalysts are expensive since their synthesis is costly. By contrast, CaO is often used as a cheap metal oxide catalyst, and it can be easily obtained from food waste. It is also an attractive catalyst for biodiesel production from bio-waste, enabling fully green routes to protect the environment.

CaO derived from waste eggshells can act as the cheapest photocatalyst for the degradation of dyes discharged into water among different oxide materials. CaO is an efficient photocatalyst and does not generate any harmful byproducts in its degradation process [16]. Worldwide, large quantities of synthetic dyes are produced via research and in different industries as colorants; out of this, some part is released into water sources, which is hazardous to nature. Dye substances are highly polluting to water and soil if they are not properly disposed of due to their low biodegradability and chemical stability, which causes serious damage to the environment [17].

Water is essential for life on Earth, but human activities are contaminating water; waste materials are released into water streams. Waste discharged from textile industries is a major source of water contamination, and this waste is an environmental threat since it is not biodegradable. Waste materials produced by textile and dye industries are harmful to the environment and living organisms [18,19]. They are difficult to degrade naturally, and special techniques are required to degrade them.

Different physical and chemical methods are used to remediate organic dyes. Physical methods like membrane filtration, electrodialysis, and adsorption are cheaper, but the maintenance of membranes and other process equipment is tedious and costly. Other chemical methods such as electrokinetic coagulation, electrochemical processes, light irradiation, chemical precipitation, adsorption, electrochemical degradation, and photochemical oxidation are used to treat textile wastewater. However, these techniques require a lot of chemicals and long processing times, consuming more energy. Moreover, they yield unwanted byproducts, and regeneration of the process is difficult, requiring high cost [17]. Most dye removal techniques are expensive and involve long processing times, and sometimes they are ineffective. By contrast, NP-based photocatalytic remediation techniques are economically viable and easy to use, and kitchen waste materials such as eggshells can be used to produce them.

Compared to all those methods, nanoparticle-mediated optical light photocatalytic dye degradation is an excellent and energy-efficient technique that can minimize the generation of toxic byproducts and the cost of the process [20]. When catalytic agents are in nanosized particles, they are very promising, showing unique and excellent physicochemical characteristics with high reactivity due to their large surface area. In addition, they are more beneficial for water treatment applications, as they have high mobility. CaO is one of the best photocatalytic degraders of organic dyes such as methylene blue, Congo red, malachite green, indigo carmine, and crystal violet [17]. Moreover, the semiconducting nature of CaO is more beneficial for photoexcitation, and also its high bandgap energy is suitable for highly energetic UV–Vis-light-activating redox reactions.

In contrast to expensive degradation techniques, photocatalytic techniques are simple and require only catalytic nanomaterials [21]. These nanomaterials can easily degrade dyes or chemical pollutants in the presence of sunlight. Some pollutants can also be degraded without the use of light. CaO is an efficient photocatalyst, and it can degrade pollutants in the absence of light; however, under light, its photocatalytic degradation capability is considerably higher. CaO NPs derived from eggshells through calcination have been used for the photocatalytic degradation of methylene blue dye to ascertain their effectiveness. Methylene blue is a cationic dye containing a conjugated aromatic moiety, and it is an environmental pollutant that can affect human health [22,23].

Chicken eggshells contain about 85–95% CaCO_3_, apart from minerals and proteins. This CaCO_3_ can easily be converted into CaO, which can be used for many purposes. For the production of CaO from eggshells, different methods such as hydrothermal approaches, the sol–gel process, thermal decomposition, precipitation, and green synthesis have been used. All these methods are time-consuming and expensive, and they involve different chemicals. Among them, green synthesis is attractive as it does not produce or require harmful chemicals. The green synthesis of nanoparticles is more cost-effective and ecologically profitable compared to chemical synthesis routes [24]. The CaCO_3_ in eggshells can be easily transformed into CaO through simple thermal treatment without the use of any chemical. This harmful-chemical-free conversion method can be considered a green synthesis route. In the calcination process, CaCO_3_ is converted to CaO and CO_2_ as follows [25]:

CaCO_3_ (s) → CaO (s) + CO_2_ (g) (1)

The major contents of eggshell CaCO_3_ can be converted into CaO through thermal decomposition at about 900 °C. Hence, compared with chemical synthesis routes, the simple calcination that is used to convert eggshells into CaO is an environmentally clean, chemical-free technique. This eggshell-based calcination is a simple and inexpensive method used to produce CaO NPs. As no chemical reagent is used in this green synthesis route, it is environmentally friendly. It provides a cheap and effective nanomaterial for pollution remediation and medical applications [26,27].

Several recycling approaches are used to reduce the volume of waste in landfills, such as fertilizers, adsorbents, soil conditioners, and additives for animal feeds, calcium supplements, and paper manufacturing. Compared to all these methods, the calcination route is advanced, permitting the formation of nanoparticles having unique properties. In this calcination technique, the CaCO_3_ is decarbonated, using-high temperature calcination to produce CaO without any chemicals. It is a useful nanomaterial for various applications, including in the field of environmental pollution remediation. Though it emits CO_2_, this level of CO_2_ emission is comparably lower than other methods and the associated manufacturing of the necessary chemical reagents. This prepared CaO can be also converted to Ca(OH)_2_, useful as a protective coating as nanolime. Simple sonication of this CaO with water and 2-propanol can form calcium hydroxide (Ca(OH)_2_). This Ca(OH)_2_ is used to conserve historical materials from deterioration and preserve cultural heritage items using nanolime coatings. Compared to other chemical routes, this eggshell-derived Ca(OH)_2_ is very cheap. The CO_2_ emissions produced by the conversion of eggshell CaCO_3_ into CaO by calcination are estimated to be about 0.79 kg of CO_2_/kg, whereas the preparation of CaCO_3_ by chemical processes produces around 0.24–0.40 kg CO_2_/kg along with 0.482–0.723 kg CO_2_/kg due to energy consumption [23,24]. This 0.79 kg of CO_2_ emission is much less than the emissions of 1.4 and 3.4 kg CO_2_/kg produced through conventional polymer preparation techniques [28]. This formed CaO can also be used to adsorb CO_2_ and form Ca(OH)_2_, useful for various applications in balancing CO_2_. As eggshells are extensively generated as waste by industries and consumers everywhere, they have become cheap raw materials.

In this calcination process, if solar or any renewable sources are used, it will be more environmentally friendly. As per recent research reports, there are calcination plants that use renewable energy sources. These fully renewable energy techniques can reduce emissions and costs also [29]. Solar thermal calcination is available and used at the industrial level at temperatures of 1350–1500 °C; if these facilities were used for the eggshell calcination process, it would be a potential techno-economic approach. Limestone calcination is conducted at high temperatures using solar power plants producing pure CaO with CO_2_ in industrial processes. These reactors can convert CaCO_3_ into CaO and CO_2_ with minimal energy, which can further be modified with CO_2_ capture facilities [30]. Moumin et al. analyzed the different scenarios of solar calciners for CO_2_ avoidance in the calcination process of solar cement plants, and they are well suited for this technique [31]. Davis et al. have also reported on using solar thermal radiation to calcinate alumina in industrial processes, which can reduce harmful radiation, including CO_2_ emissions [32]. Panagiotou et al. used calcined eggshells for phosphorous removal by adsorption. This ability of phosphorous removal by calcined eggshells is valuable [33]. Therefore, this calcination of waste eggshells is a potential technique to mitigate environmental problems. Moreover, CaCO_3_ obtained from eggshells is highly pure; it does not require any chemical reagents, making it a chemical-free process.

In this paper, the successful preparation of green CaO through the decomposition of chicken eggshells and its characteristics are reported. Furthermore, its potential for use in antibacterial and photocatalytic applications is demonstrated.

## 2. Experimental Details

**Figure 1:**
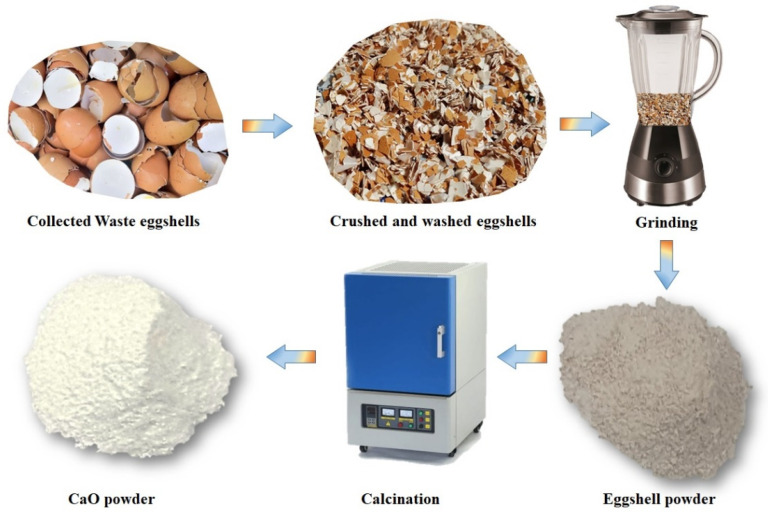
Schematic representation of eggshell-derived CaO preparation.

The typical experimental procedure followed for the preparation of eggshell-derived CaO is schematically shown in Figure 1. Chicken eggshells were collected from kitchen waste, crushed into small pieces, and washed several times with deionized (DI) water to remove unwanted residues. The washed eggshells were dried at room temperature for two days, and they were then ground to fine powder in a mixer grinder. This eggshell powder obtained was calcined at 900 °C for 1 h. Subsequently, it was cooled to around 100 °C, and the resulting CaO powder was crushed using a pestle and mortar to fine powder with a nanoscale particle size, as shown in Figure 1. The resulting CaO NPs were collected and preserved for further characterization and use. They were characterized using powder X-ray diffraction (XRD) (Bruker, Billarica, MA, USA), scanning electron microscopy (SEM) (Hitachi S-4800, Tokyo, Japan), transmission electron microscopy (TEM), Fourier transform infrared (FTIR) spectroscopy, ultraviolet–visible (UV–Vis) spectroscopy, and X-ray photoelectron spectroscopy (XPS) (ULVAC, Chigasaki, Japan). Optical-illumination-dependent current–voltage (I–V) measurements were performed by coating the prepared CaO nanoparticles on a p-Si substrate using a Keithley 2611B source meter (Cleveland, OH, USA) at different illuminations. Furthermore, their potential for use as an antibacterial agent and as a photocatalyst was investigated. The converted CaO NPs were also analyzed by cyclic voltammetry to understand the catalytic effectiveness of the eggshell-derived CaO.

### 2.1. Antimicrobial Activity

The antibacterial potency of eggshell-derived CaO NPs against the Gram-positive and Gram-negative bacteria *Staphylococcus aureus* and *Escherichia coli*, respectively, was assessed by performing a disc diffusion experiment [34]. For the preparation of bacterial suspensions, both strains were cultivated in Luria–Bertani (LB) broth at 37 °C for 24 h (180 rpm) on LB agar plates. The bacterial suspensions (1 × 10^6^) were then distributed on LB agar plates using a sterile glass spreader. Sterile filter paper discs (6 mm in diameter) were placed on the infected plates along with sterile water as a negative control (NC), ampicillin as a positive control (PC), and sterile filter paper discs with different CaO concentrations (10, 20, 30, and 40 µg/mL). All plates were incubated for 24 h at 37 °C. After incubation, the dimensions of the inhibitory zone that formed during incubation with various CaO concentrations were calculated.

### 2.2. Photocatalytic Study

The photocatalytic activity of the prepared CaO NPs was investigated by examining the degradation of methylene blue (MB) dye by the NPs. A stock solution of MB was prepared by dissolving 50 mg of MB in 100 mL of DI water. Different concentrations of CaO (5, 15, and 20 mg) were dispersed in 20 mL aliquots of the MB stock solution by stirring. MB solutions with different CaO concentrations were exposed to UV light and direct sunlight separately at different times under ambient conditions. Subsequently, they were analyzed using UV–Vis absorption spectroscopy to obtain the degradation levels. A UV lamp of 75 µW was used in this photocatalytic experiment. For sunlight irradiation, direct sunlight at about 1 p.m. on a clear day was used. The photocatalytic activity of CaO was obtained by measuring the amount of absorbance (A). The degradation efficiency of CaO was calculated as

(2) $$Photocatalytic\;efficiency\;\left(\%\right)=\frac{A_{0}-A_{t}}{A_{0}}\times 100$$

where *A*_0_ is the initial absorbance of the MB dye and *A_t_* is the absorbance of the dye after light irradiation for time *t*.

## 3. Results and Analysis

### 3.1. Structural and Chemical Analysis

**Figure 2:**
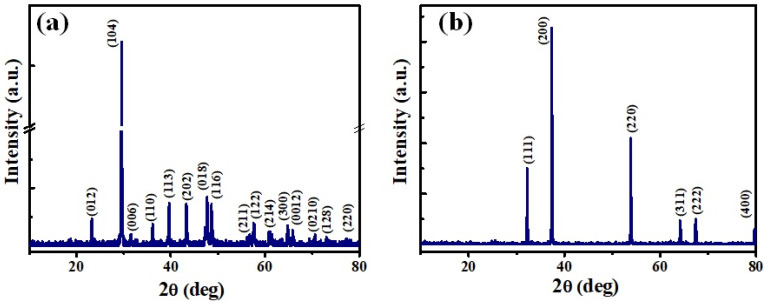
X-ray diffraction patterns of eggshell powder: (**a**) before calcination and (**b**) after calcination.

The high-temperature calcination of raw eggshells at about 900 ◦C converted them to pure CaO through the vaporization of water molecules and the decomposition of CaCO_3_ and Ca(OH)_2_. The CaO NPs obtained were characterized by determining their structural, chemical, optical, and morphological properties. X-ray diffraction patterns of the eggshells before and after calcination are shown in Figure 2a,b. The XRD pattern of the raw eggshells showed that CaCO_3_ matched well with the calcite phase of CaCO_3_ (PDF Card No. 00-081-2027). It showed peaks at 23.25, 29.55, 31.54, 36.12, 39.52, 43.45, 47.64, 48.6, 56.62, 57.5, 61, 64.8, 65.7, 70.29, 72.96, and 76.47 corresponding to the (012), (104), (006), (110), (113), (202), (018), (116), (211), (122), (214), (300), (0012), (0210), (128), and (220) planes of the calcite phase of CaCO_3_, respectively, with the main peak being that at 29.55. Figure 2b shows the XRD peaks of the eggshells after calcination. The peaks at 32.3, 37.4, 54, 64.2, 67.5, and 79.8 correspond to the (111), (200), (220), (311), (222), and (400) planes of the CaO NPs, respectively (PDF Card No. 99-0070). The sharp and high intense peaks suggest highly polycrystalline CaO NPs [13,35]. Furthermore, they confirm the complete conversion of CaCO_3_ in the raw eggshells into CaO through the chemical-free calcination treatment. The crystallite size (*d*) of the converted CaO NPs was calculated using the Scherrer equation (Equation (3)), and it was found to be 59 nm. The lattice strain (*ε*) and dislocation density (*δ*) of the crystallites were also calculated using Equations (4) and (5), and they were obtained to be 3.56 × 10^−3^ and 2.85 × 10^−3^, respectively [36].

(3) $$d=\frac{k\lambda }{\beta \;cos\theta }$$

(4) $$\varepsilon =\frac{\beta }{4\;tan\theta }$$

(5) $$\delta =\frac{1}{D^{2}}$$

Here, *k* is the Scherrer constant (=0.96), *λ* is the wavelength (=1.54 Å) for Cu Kα, *β* is the full width at half maximum of the peak, and *θ* is Bragg’s diffraction angle.

**Figure 3:**
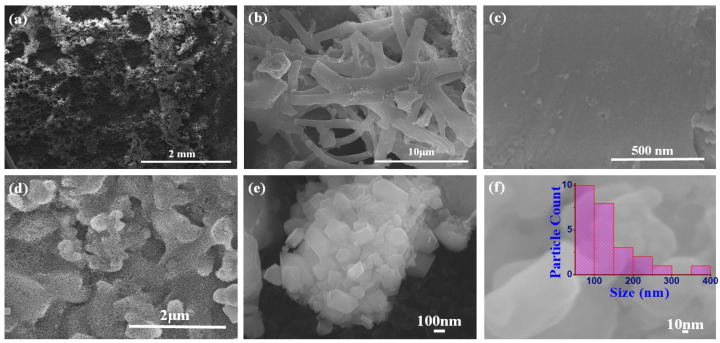
FE-SEM images of eggshell-derived CaO powder: (**a**–**c**) before calcination and (**d**–**f**) after calcination. Inset of (**f**) shows particle size distribution.

**Figure 4:**
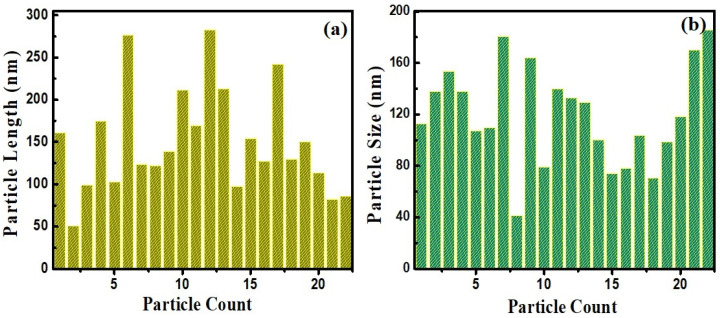
Size analysis of CaO nanoparticles showing different (**a**) lengths and (**b**) breadths.

**Figure 5:**
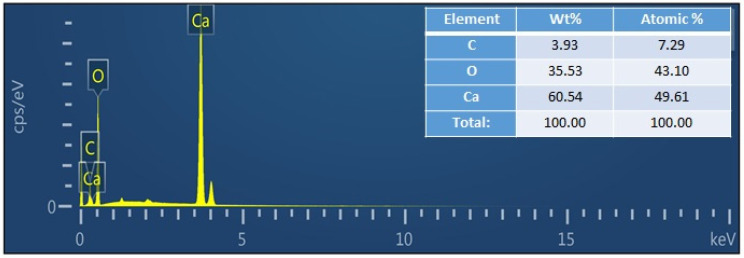
EDAX result for the eggshell-derived CaO nanopowder.

The surface morphologies of the produced eggshell powder and CaO NPs were studied using field-emission scanning electron microscopy (FE-SEM), and the FE-SEM images are shown in Figure 3. Figure 3a–c show images of the eggshell powder before calcination. Porous multi-linked rod-shaped CaCO_3_ particles with lengths exceeding 10 μm are apparent. Figure 3d–f show SEM images of the CaO NPs obtained after calcination. Perfect hexagonal, agglomerated CaO nanorods of different sizes (Figure 3e) can be seen, and they indicate a perfect crystalline nature. The particle size distribution plot in the inset of Figure 3f shows that most of the rods were nanosized with different lengths and breadths. Figure 4 shows length and breadth distribution of the CaO nanoparticles, indicating rods of different sizes. The length of the particles varied from 50 nm to 275 nm, showing a majority of the particles in between 50 nm and 100 nm (Figure 4a), and most of them were thicker, showing more than 100 nm breadth. The calcination treatment converted CaCO_3_ into regular-shaped CaO particles with improved crystalline structure [37]. The energy-dispersive X-ray analysis (EDAX) result obtained to analyze the composition of the product is shown in Figure 5. It indicates the presence of Ca and O along with a small amount of C originating from the solvent and sample holders, confirming the formation of CaO from the eggshells, which is consistent with the XRD results [13].

**Figure 6:**
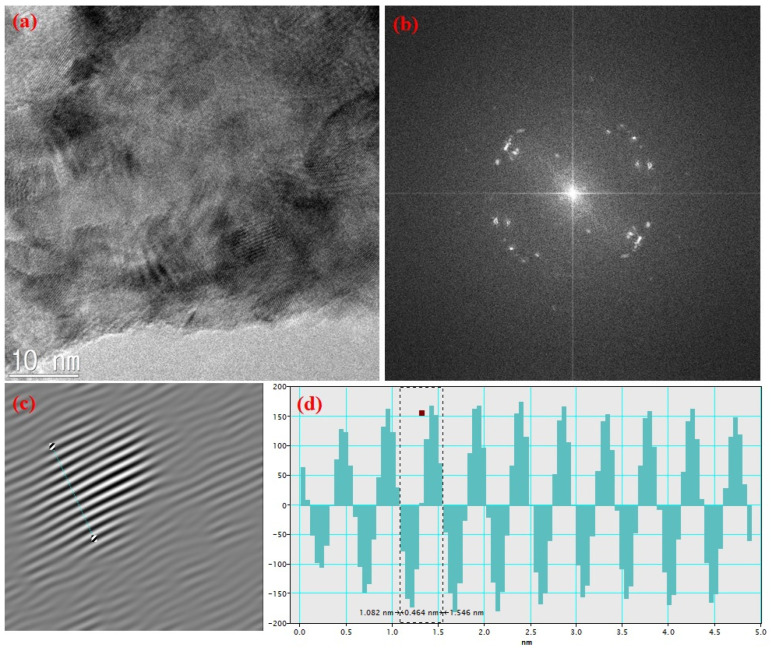
(**a**) TEM image of CaO NPs, (**b**) SAED pattern, (**c**) FFT fringe pattern, and (**d**) interplanar distance.

The converted CaO NPs were also studied by TEM to examine their microstructural properties. Figure 6a shows a highly magnified TEM image of CaO nanoparticles, indicating their crystalline nature from the fringe patterns. Their SAED patterns and interplanar distances, calculated using Gatan software (GMS 3), are shown in Figure 6b–d. The spot-like pattern of the SAED pattern justifies the crystalline purity of the prepared CaO NPs. Interplanar distances of 0.464 nm and 0.243 nm were obtained as shown in Figure 6d, calculated using two different dots of SAED patterns, which are in agreement with previous reports on CaO [38,39].

**Figure 7:**
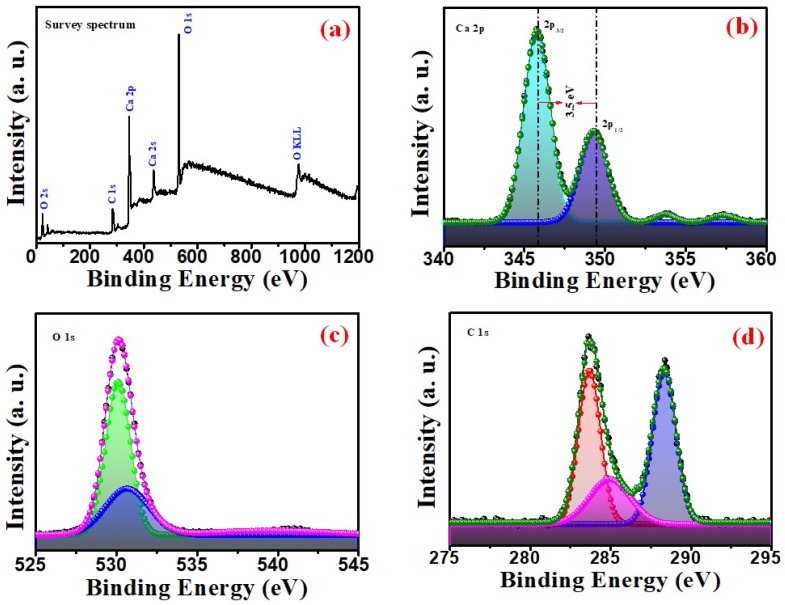
XPS spectra of CaO NPs (**a**) full survey, (**b**) Ca 2p, (**c**) O 1s, and (**d**) C 1s.

The elemental composition of the prepared CaO was confirmed using XPS. The XPS spectrum is displayed in Figure 7, indicating Ca and O related peaks of Ca 2s, Ca 2p, and O 1s states in the survey spectrum (Figure 7a). The deconvoluted high-resolution spectra of Ca 2p and O 1s are shown in Figure 7b and Figure 7c, respectively, and these energy values are also in accordance with previous reports [40,41]. The spin–orbit-coupling-related peaks of 2p_3/2_ and 2p_1/2_ of the Ca 2p spectrum exhibit a separation of 3.5 eV. This separation energy of the Ca^2+^ oxidation state is exactly equal to CaO (i.e., 3.5 eV), indicating complete conversion of the eggshells into pure crystalline CaO.

The deconvoluted peaks of the O 1s spectrum shown in Figure 7c exhibits two peaks at approximately 530 eV and 531 eV with the oxygen in the CaO NPs. Figure 7d shows the fitted C 1s spectra with peaks at 285 eV, 286 eV, and 288 eV, assigned to C–C, C–O, and C=O vibrations, respectively, due to the association of carbon with CaO [40]. As there are no C-related peaks in the XRD results, this C trace indicates adsorbed carbon from solvents and atmosphere due to the highly reactive nature of CaO with its ambient environment.

### 3.2. Optical Characterization

**Figure 8:**
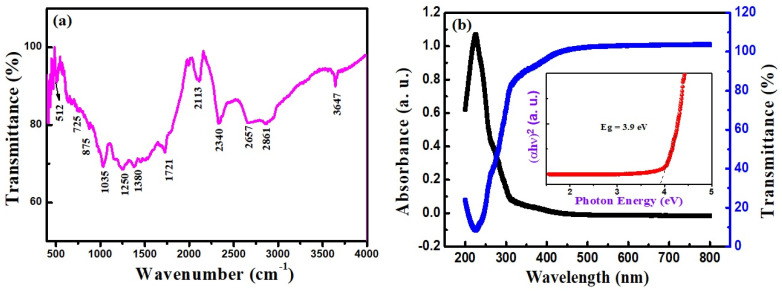
(**a**) FTIR transmittance and (**b**) UV–Vis absorbance and transmittance spectra of eggshell-derived CaO NPs (inset: Tauc plot).

The eggshell-derived CaO NPs were also characterized using a PerkinElmer FTIR spectrometer. FTIR characterization is useful to qualitatively identify the chemical constituents of a compound at molecular vibrational frequencies. Figure 8a shows the FTIR spectrum of the eggshell-derived CaO NPs. The broad band centered around 1400 cm^−1^ corresponds to the C–O bond of the eggshells; other characteristic bands are apparent at 512, 875, 1035, 1721, 2113, 2340, 2657, 2861, and 3647 cm^−1^, and they indicate the eggshell-derived CaO [35]. The peak at 2340 cm^−1^ represents the N-H bond of amines and amides in the eggshells. Bands observed at 1035 and 875 cm^−1^ are ascribed to the C–O bond of CaO. The peak at 3647 cm^−1^ is attributed to the O–H bond produced from the water molecules present on CaO NPs, and the peak at 2340 cm^−1^ is due to adsorbed atmospheric CO_2_. The Ca–O vibration bond can be identified at 512 cm^−1^. The presence of the peak at the position of 875 cm^−1^ is due to the out-of-plane bending mode of carbonate groups. The wide bonds observed around 1400 cm^−1^ and 2700 cm^−1^ point to the presence of carboxylic and hydroxyl groups along with amines and amides. These phytoconstituents can enhance the stability of CaO NPs [36]. The UV–Vis absorbance and transmittance spectra of the CaO NPs recorded in the wavelength range of 200 nm to 800 nm are shown in Figure 6b. An absorbance edge is visible around 225 nm. After 225 nm, the absorbance decreases, and it is almost zero in the visible region after 400 nm. The bandgap energy calculated from the Tauc plot (Figure 8b inset) was obtained to be about 3.9 eV, which is lower than that of bulk CaO [42]. This smaller bandgap of CaO NPs is comparable with previously reported values [43]. The decrease in the optical bandgap energy of the eggshell-converted CaO may be associated with the presence of defect states in the energy gap and structural modifications [44].

### 3.3. Optical Sensing Property

**Figure 9:**
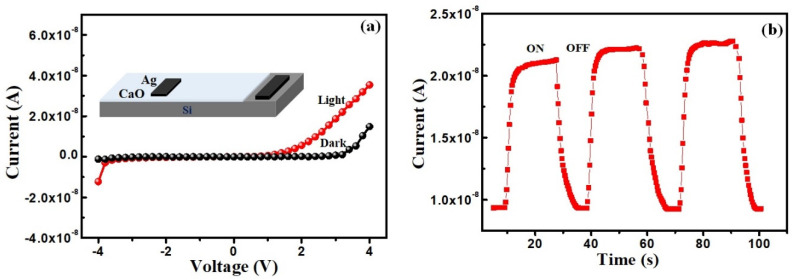
(**a**) Current–voltage plots of CaO NPs/Si heterostructure and (**b**) current vs. time plot of the device under on and off conditions for white light illumination.

Optical detection property of the eggshell-converted CaO was also studied by forming a heterostructure with a p-Si substrate, as shown in the inset of Figure 9a. This optical sensing is also a very useful property of oxide materials for a variety of applications. Figure 9a shows the current–voltage (I–V) plots of the CaO NPs/Si heterostructure under dark and white light illuminated conditions. The non-linear asymmetric nature of the I–V response indicates the formation of a Schottky diode between the CaO and p-Si substrate. The very high turn-on voltage around 3 V indicates high barrier height formation. However, the irradiation of the white light current is increased, showing photo-induced electronic excitation. Optical switching of the CaO/p-Si device was also studied, as shown in Figure 9b. It shows a good sensing effect of the heterostructure with good response and recovery of the device. Compared to the optical response at illumination, the recovery after the light switch-off is small. Though the bulk CaO is electrically insulating in nature, the nanostructured CaO particles showed improved electrical properties useful for opto-electronic applications.

### 3.4. Photocatalytic Study

**Figure 10:**
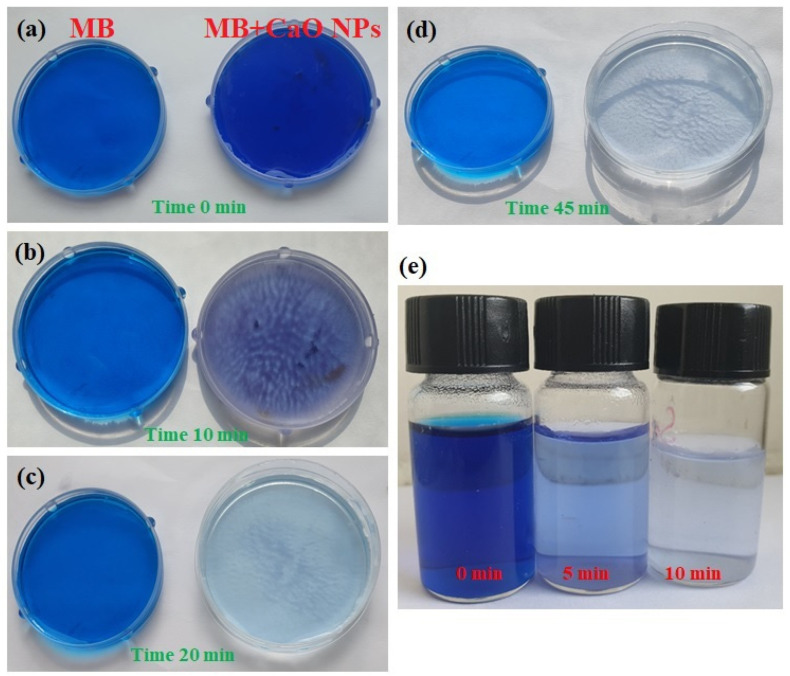
Photodegradation of MB dye by CaO NPs for exposure to (**a**–**d**) sunlight and (**e**) UV light for different durations.

The photocatalytic response of the prepared CaO NPs was evaluated by examining the photodegradation of aqueous MB dye by the NPs. Initially, CaO NPs dispersed in the MB solution were exposed to sunlight for 15 min, and their color was observed to detect any change; three dispersions with different CaO NP concentrations (5, 10, and 20 mg of CaO in 20 mL aliquots of the stock solution) were considered. Among the three dispersions, the one with 20 mg of CaO showed a clear color change. After this observation of the photocatalytic activity of CaO on the MB dye, the experiment was repeated with dispersions containing 20 mg of CaO NPs for exposure to sunlight and UV light for different durations. Figure 10 shows the color change of a CaO NP-dispersed MB dye solution that was exposed to both UV light and direct sunlight for different intervals. Under illumination by both sunlight (Figure 10a–d) and UV light (Figure 10e), the change in the color of the MB solution depended on the exposure duration (10, 20, 30, and 45 min). A colorless solution was obtained after 45 min of sunlight exposure, whereas, in the case of UV light exposure, the solution became colorless within 10 min. For sunlight exposure, the CaO NP-dispersed MB solution was kept outdoors in sunlight without being disturbed.

**Figure 11:**
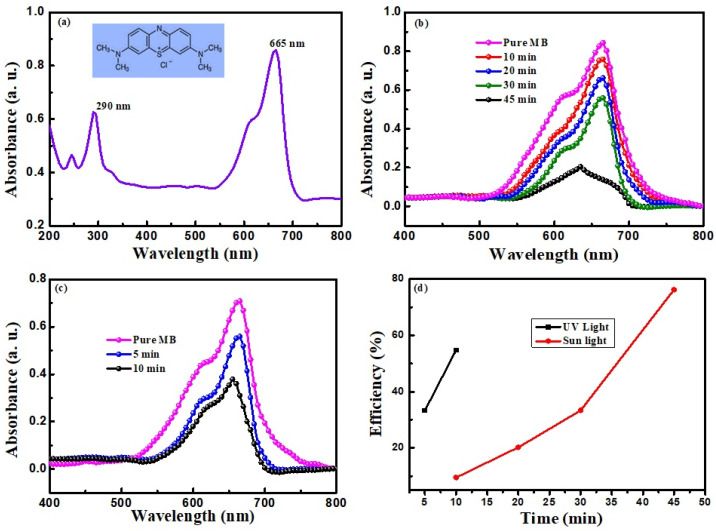
Photodegradation of MB dye by CaO NPs and the UV–Vis absorption spectrum of (**a**) pure MB, (**b**) CaO NP-mixed MB for different exposure times for sunlight, and (**c**) UV light; (**d**) the catalyst efficiency vs. time plot.

UV–Vis absorption spectra were obtained for MB-CaO mixed solutions exposed to both UV and sunlight for different durations to validate the observation of the color changes. The different UV–Vis spectra obtained are shown in Figure 11. Figure 11a shows the absorption spectrum of MB in the range of 200 nm to 800 nm. Two peaks can be observed at 290 and 665 nm, with increased absorbance at about 665 nm [23]. Hence, the wavelength range of 400–800 nm was considered to measure the absorbance of the light-exposed solutions, as shown in Figure 11b,c. Figure 11b shows the UV–Vis spectra of the solution obtained after 10, 20, 30, and 45 min of exposure to sunlight. Similarly, solutions exposed to UV light for 5 and 10 min are shown in Figure 11c. The UV–Vis spectra obtained for the CaO NP-dispersed MB solution after exposure to both UV light and sunlight showed a lower intensity of the absorption peak, indicating the photocatalytic degradation of the MB dye. The further decrease in absorption for an increased time of exposure indicates the effectiveness of the photocatalyst in the degradation process. This efficient and short-time photodegradation ability indicates a large surface area due to the porosity of the CaO NPs and a large number of free radicals produced on the CaO NPs [23]. The increased catalytic activity of CaO with UV light indicates more redox reactions resulting from more electron–hole pairs formed because of the high energy of UV light and the suitable bandgap of the produced CaO.

**Figure 12:**
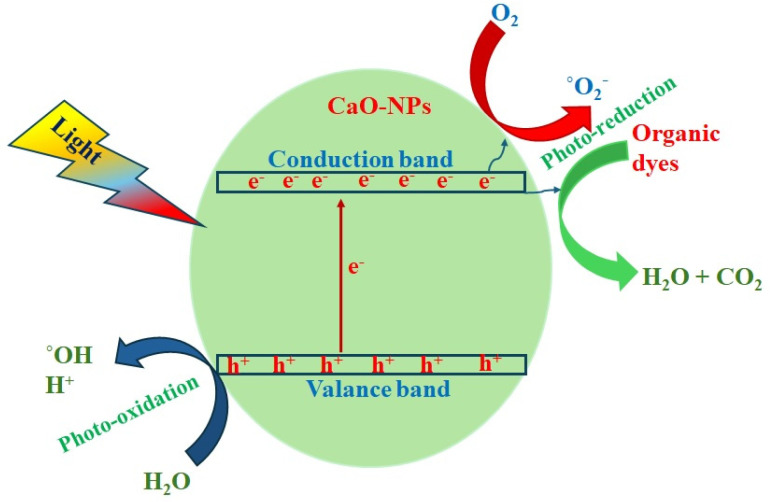
Schematic of photodegradation process of CaO NPs.

The mechanism of photocatalytic dye degradation is schematically explained in Figure 12. When the light of sufficient energy equal to the energy gap of CaO is incident, the electrons in the valance band are excited to a conduction band forming free electrons, and simultaneously, holes are created in the valance band. These released electrons react with the dissolved oxygen (O_2_) in the aqueous solution and form active free radicals (.OH and O_2_^−^). Simultaneously, the holes also react with the dissolved products and produce free radicals. These free radicals react with dye molecules and their functional groups both by photo-induced oxidation and reduction processes, breaking the molecular chains causing de-coloration, as shown in the Figure 12 [23].

The produced holes (h^+^) react with electron (e^−^) donors in the solution and generate free radicals, which oxidize the organic dyes on the surface. During this process, CO_2_, water, and other organic products are formed [45]. The following equations describe the photocatalytic reactions produced in the the catalytic system for free radical production:

(6) $$CaO+h\nu \to CaO+e_{CB}^{-}h_{VB}^{+}$$

(7) $$h_{VB}^{+}+{OH}^{-}\to {OH}^{\cdot }$$

(8) $$h_{VB}^{+}+H_{2}O\to {OH}^{\cdot }+H^{+}$$

Chicken eggshells can provide many advantages as adsorbents to remove contaminants in water, with affordable costs using only pulverization and calcination to convert as CaO NPs [4]. The semiconduction nature of CaO is more beneficial for photoexcitation, and also its high bandgap energy is suitable for highly energetic UV–Vis-light-activating redox reactions.

### 3.5. Electrocatalytic Study

**Figure 13:**
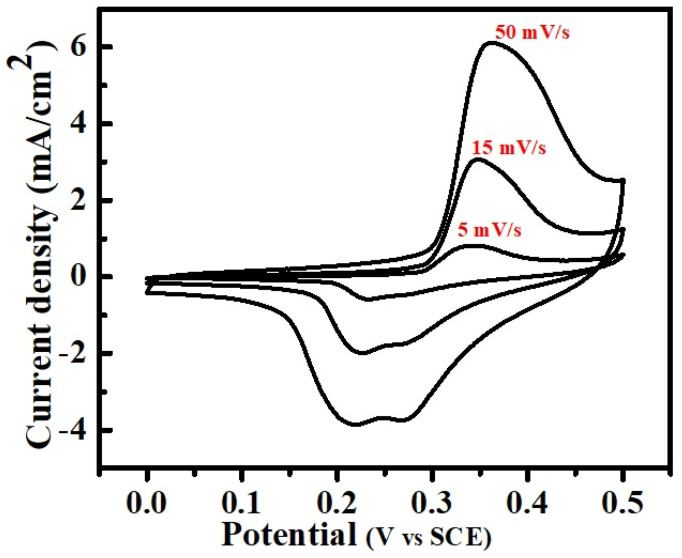
Cyclic voltammetry curves of CaO NPs at different scan rates.

The cyclic voltammogram (CV) study conducted for the CaO NPs to analyze the catalytic performance is shown in Figure 13. The CV curves were recorded for the voltage range between 0 and 0.5 V vs. SCE at different scan rates using a standard three-electrode cell setup with Pt and Ag/AgCl as the counter and reference electrode, respectively, in a 2 M KOH aqueous solution. The clear and well-aligned loops of the CV curves with distinct redox peaks of all scan rates signifies the good and stable catalytic performance of the CaO as an electrode material, which indicates the rapid and reversible charge transfer characteristics. The increase in redox peaks with the increased scan rate indicates a steady and fast redox reaction taking place between the electrolyte and electrode surface. The rectangular-shaped CV curves with well-defined symmetrical redox peaks suggest battery-type storage properties of the materials. The scan-rate-dependent changes in the reduction and oxidation peaks reveal that at high scan rates, the CV curves display small double peaks, while at low scan rates, double peaks appear in the oxidation states. This confirms the mixed state of pseudocapacitive and Faradaic redox reactions.

### 3.6. Antibacterial Activity of CaO NPs

**Figure 14:**
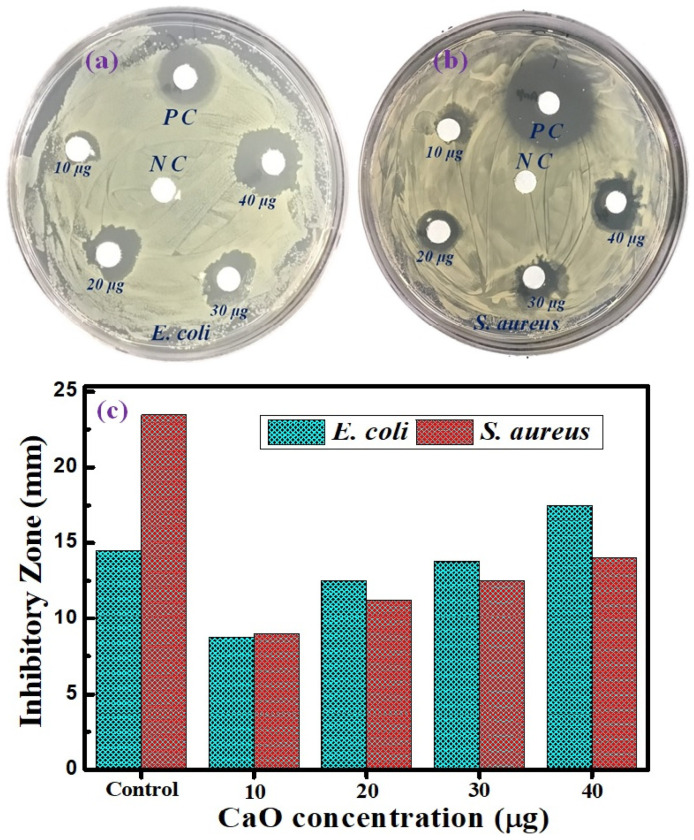
(**a**) Antibacterial inhibitory zone of CaO NPs for (**a**) *E. coli* and (**b**) *S. aureus* for different CaO concentrations and (**c**) comparative plots of *E. coli* and (**b**) *S. aureus* inhibition levels with positive control.

**Figure 15:**
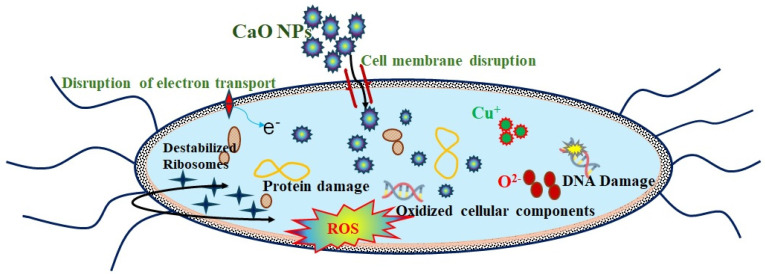
Schematic representation of CaO NP antibacterial activity.

The antibacterial activity of the prepared CaO NPs against *E. coli* and *S. aureus* was examined. Antibacterial activity with the formation of clear inhibition zones around the CaO NP-loaded discs was observed for both bacterial strains. The antibacterial activities of the CaO NPs at different concentration levels against *E. coli* and *S. aureus* are shown in Figure 14. The antibacterial activity of CaO NPs against *E. coli* is depicted in Figure 14a. A large inhibitory zone of *E. coli*, measuring 14.5 mm, is clearly visible for ampicillin, which was used as a positive control (PC), while there was no activity in the negative control (NC), with a 0 mm inhibition zone. A 17.5 mm inhibitory zone was seen against *E. coli* at the highest dose of CaO NPs (40 µg/mL), indicating that the NPs inhibited *E. coli* growth more efficiently than ampicillin. Significant bactericidal activity was also seen for the other concentrations, namely 10 (9 mm), 20 (12.5 mm), and 30 µg/mL (14 mm). *S. aureus* was more severely inhibited by ampicillin than *E. coli* (Figure 14b)*. S. aureus* was also inhibited significantly by CaO NPs, with the extent of inhibition depending on their concentration level (10 µg/mL: 9 mm; 20 µg/mL: 11.5 mm; 30 µg/mL: 12.5 mm; and 40 µg/mL: 14 mm). The CaO NPs, however, more significantly inhibited the development of *E. coli* compared with *S. aureus.* Because Gram-negative bacteria are more easily affected by NPs than Gram-positive bacteria owing to the presence of a thin peptidoglycan layer on the cell wall of Gram-negative bacteria, the CaO NPs easily penetrated the thin wall, entered the cell, and disrupted the bacterial cell function and growth [46]. The synthesized NPs can produce reactive oxygen species, such as hydrogen peroxide and superoxide radicals, that interact with bacterial cells and cause oxidative stress, which kills bacteria by causing DNA damage, protein denaturation, and lipid peroxidation [47]. Another way to inhibit bacterial activity is to disrupt the phospholipid membrane by inducing the hydroxyl radical to react with it [48]. The antibacterial activity of nanoparticles depends on their size and concentration. Formed cations and anions interact with the respective charged components of microbial cells, and as a result micromicrobes are collapsed and are not able to proliferate and sustain. The antimicrobial process of CaO NPs is depicted in Figure 15. This study clearly observed that the CaO NPs showed a significant bacterial growth inhibition zone when applied to both Gram-negative and Gram-positive bacteria, which indicates the high potential of the NPs for use as an antibacterial agent. The antibacterial levels of both bacteria are shown in Figure 14c in comparison with ampicillin. The observed results agree well with previous reports on antibacterial activities of CaO NPs. The inhibition zones produced clearly show the biocidal properties of the eggshell-derived CaO NPs. As the NPs have a large surface area, they can easily penetrate through the cell membrane by adhering to bacterial surfaces. The resulting damage of cell walls causes the leakage of intracellular matter, leading to the death of the bacteria.

## 4. Conclusions

Waste eggshells were successfully converted into CaO NPs through simple calcination without using any chemical. The CaO produced could be termed green-synthesized CaO. The various characterizations of the eggshell-derived CaO showed that it comprised rod-shaped crystalline CaO NPs. This green-synthesized CaO showed good photocatalytic degradation of MB dye, with 76% degradation for 45 min of exposure to sunlight. It showed higher efficiency for UV light exposure, with a degradation of 55% for 10 min of exposure. The prepared CaO NPs also had good antibacterial activity, comparable to that of ampicillin (an antibacterial agent). They showed good antibacterial activity against Gram-positive and Gram-negative bacteria, namely *S. aureus* and *E. coli*, respectively, with higher efficiency for the Gram-negative bacteria. An inhibitory zone of 17.5 mm was formed against *E. coli* for a CaO dose of 40 µg/mL, while a 14.5 mm inhibitory zone was formed for ampicillin. This showed the higher efficiency of the CaO NPs. These prepared CaO NPs also showed good catalytic performance suitable for electrochemical applications. Notably, the green-synthesized CaO has the potential to be used for effective environmental remediation.

## Data Availability

The raw data supporting the conclusions of this article will be made available by the authors on request.
